# An Experimental Analysis of the High-Cycle Fatigue Fracture of H13 Hot Forging Tool Steels

**DOI:** 10.3390/ma15217411

**Published:** 2022-10-22

**Authors:** Erik Calvo-García, Sara Valverde-Pérez, Antonio Riveiro, David Álvarez, Manuel Román, César Magdalena, Aida Badaoui, Pedro Moreira, Rafael Comesaña

**Affiliations:** 1 Materials Engineering, Applied Mechanics and Construction Department, E.E.I., University of Vigo, 36310 Vigo, Spain; 2 LaserOn, CINTECX—Research Center in Technologies, Energy and Industrial Processes, University of Vigo, Lagoas-Marcosende, 36310 Vigo, Spain; 3 ENCOMAT, CINTECX—Research Center in Technologies, Energy and Industrial Processes, University of Vigo, Lagoas-Marcosende, 36310 Vigo, Spain; 4 CIE Galfor (CIE Automotive Group) P.I. San Cibrao das Viñas, 32901 Ourense, Spain; 5 INEGI—Institute of Science and Innovation in Mechanical and Industrial Engineering, Rua Dr. Roberto Frias, 400, 4200-46 Porto, Portugal

**Keywords:** high-cycle fatigue, corrosion fatigue, hot forging tool steels, AISI H13, failure analysis

## Abstract

In this study, the axial fatigue behaviour of hot forging tool steels at room temperature was investigated. Fatigue tests were performed on two steels within the same H13 specification. The fatigue tests were carried out in the high-cycle fatigue domain under normal conditions. These tests were also performed on specimens in contact with a corrosive medium, applying stress values that led to the high-cycle fatigue domain under normal conditions for the sake of comparison. Both materials showed similar fatigue strengths when they were tested under normal conditions. In contrast, corrosion fatigue lives were much lower than in normal tests and differed significantly between the two steels. Crack initiation was triggered by microstructural and surface defects in the normal tests, whereas the formation of corrosion pits caused crack initiation in the corrosion fatigue tests. Moreover, a fracture surface analysis revealed dissimilar crack propagation areas between both steels, which suggested that both steels had different fracture toughness. These results were in line with the differences observed between the carbide and grain sizes of both of the material microstructures.

## 1. Introduction

Hot forging tool steels present excellent mechanical properties, such as extraordinary yield and tensile strengths even at high temperatures [1,2] and a fairly good balance between hardness and fracture toughness when they are subjected to an appropriate thermal treatment [3]. Hence, these steels are extensively used for manufacturing forging dies, pressure die casting tools, and extrusion tools [4]. However, in spite of their outstanding mechanical properties, these steel tools suffer from severe damage when they are subjected to thermal [5], mechanical [6], and tribological [7] fatigue loads, which cause the initiation and propagation of cracks and eventually the fracture of these tools [8]. The fracture of hot forging tools is a crucial issue for many industrial sectors since it requires large annual investment in maintenance and tool replacement [9]. In order to reduce these costs and maximise the replacement times of forging tools, a fundamental understanding of the fatigue strength and the fatigue fracture mechanisms of hot forging tool steels is required.

The fatigue behaviour of hot forging tool steels depends strongly on their mechanical properties, especially on the hardness and the fracture toughness. The surface hardness of a material improves its fatigue strength, as a higher hardness results in a higher resistance to the local plastic deformation required to open a crack [10]. Since fatigue cracks usually start at the surface of a component, a high surface hardness delays crack initiation and thus extends the fatigue life of the component [11]. On the other hand, the fracture toughness provides the resistance to support loads in the presence of cracks or defects, so a higher fracture toughness improves the fatigue crack propagation behaviour of the material.

The mechanical properties of hot forging tool steels can be varied substantially by modifying the variables of the heat treatment they are subjected to. The heat treatment of these steels often consists of a quenching stage followed by two or even three tempering stages. The effects of the quenching stage are controlled by the selection of an austenitising temperature. An increase in the austenitising temperature promotes grain growth, which causes a decrease in the yield strength (YS) and ultimate tensile strength (UTS) of the steel according to the Hall–Petch equation [12]. Moreover, higher austenitising temperatures also favour the dissolution of primary carbides that precipitate during the tempering stages as smaller secondary carbides, thus enhancing the hardness of the steel [13]. As for the tempering stages, it has been observed that higher tempering temperatures result in lower values of the UTS and the hardness, whereas the fracture toughness is improved [14]. During tempering, a softening process takes place where the martensite microstructure decomposes and carbides precipitate [4]. The presence of smaller carbides and their homogeneous distribution is beneficial for the fracture toughness of the steels [3].

Most previous investigations into the fatigue behaviour of hot forging tool steels have aimed to characterise the performance of these alloys in the low-cycle fatigue (LCF) domain [15,16]. However, the results of the high-cycle fatigue (HCF) characterisation of hot forging tool steels remain scarce in the literature. Shinde et al. [17] performed rotating bending HCF tests on conventionally heat-treated H13 steel. As a result of these tests, a fatigue strength of 566 MPa was obtained at 10^7^ cycles. Likewise, Korade et al. [18] evaluated the fatigue behaviour of H21 steel using rotating bending HCF tests up to 10^7^ cycles. The resultant fatigue strength for conventionally heat-treated specimens was found to be 560 MPa. 

Work environments can worsen the fatigue behaviour of steels by causing corrosion damage. For instance, Papageorgiou et al. [19] analysed the failure mechanisms of an H13 steel hot forging die that failed earlier than expected during its operation. This failure analysis revealed that the cooling agent utilised in the working process of this die presented a high salt concentration and caused a severe corrosion attack, which increased the surface roughness of the die and thus accelerated the damage process. The corrosion fatigue phenomenon can be triggered by many factors, such as contact with water, condensation of moist air, or adsorption of gases [20]. Therefore, understanding the interaction between corrosive environments and fatigue performance is also important to guarantee a high durability of hot forging tool steel components. 

Numerous investigations have dealt with the corrosion fatigue analysis of various types of steels. Yongmei et al. [21] performed axial fatigue tests on maraging steel specimens within a chamber filled with a NaCl solution. The maraging steel showed a dramatic decrease in the number of cycles to failure in the tests carried out in saline bath tests compared to conventional fatigue tests. Amongst these results, it was also observed that a lower loading frequency and a lower stress ratio decreased the fatigue lives of the specimens. Ebara [22] analysed the effect of the corrosive medium concentration on the corrosion fatigue results of a stainless steel, observing that a higher concentration of NaCl in the corrosive medium affected fatigue strength and crack growth rates negatively. May et al. [23] evaluated the failure mechanisms of a martensitic stainless steel submerged in a saline solution. The origin of cracks was attributed to the rupture of local passive films in the surface of the tested specimens, leading to an increased corrosion attack that favoured crack initiation. Nevertheless, no results regarding the corrosion fatigue of hot forging tool steels have yet been presented in the literature.

The purpose of this work was to analyse the fatigue behaviour and fracture mechanisms of AISI H13 steel, which is one of the most common hot forging tool steels. This analysis included a study of the dissimilar effects of the microstructure on the results of the corrosion fatigue (CF) tests when compared with the results of the conventional fatigue tests. In order to achieve this goal, a microstructural analysis was performed on H13 samples from two different manufacturers that underwent analogous heat treatments. Hardness and tensile tests were carried out on both steels, since mechanical properties have an important influence in the fatigue behaviour. Next, axial fatigue tests were performed at room temperature in the HCF domain under normal conditions. Using a saline solution as a corrosive medium and applying the same stress values that led to HCF failure under normal conditions, CF tests were carried out. Finally, fracture surfaces were analysed to identify the mechanisms that led to the failure of the specimens.

## 2. Materials and Methods

### 2.1. Materials

The materials analysed here were two hot forging tool steels from different manufacturers within the same AISI H13 specification. H13 steels were selected to carry out this research as this specification is one of the most utilised for hot forging tool manufacturing. The H13 designation is also referred to in the literature as 1.2344 or SKD61 [24]. The chemical compositions in weight percentages of both the H13 steels, namely A and B, are displayed in Table 1. It can be observed that both steels have considerably similar chemical compositions and that H13 steels have high contents of chromium, molybdenum, and vanadium. The content of chromium increases the resistance to oxidation and high temperatures, whereas molybdenum improves the hardenability of the steel and vanadium enhances its strength and toughness [25].

Both H13 steels were manufactured using an electro-slag remelting (ESR) process. The ESR process has been demonstrated to be an effective process for obtaining high-strength steels with a high degree of cleanliness, smaller inclusions, and enhanced fatigue strengths [26]. According to the assessment of the statistical influence of the ESR process on the fatigue behaviour of H13 steels, it was proved that fatigue lives were significantly enhanced for the refined steel specimens [27].

The H13 steels analysed in this study were obtained from actual crankshaft forging dies. These dies were austenitised, quenched in water, and then tempered twice to attain a hardness of 46 HRC. The A-steel forging die was austenitised to 1025 °C for 2.5 h and tempered twice at temperatures of 570 °C and 605 °C for 4 h and 5 h, respectively. Similarly, the B-steel forging die was austenitised to 1020 °C for 2.5 h and tempered twice for 4 h and 5 h at a temperature of 600 °C. The heating procedure during the stages of the heat treatments was adapted in order to ensure a homogeneous distribution of temperature in the whole of the forging dies. Such recommended heat treatment procedures for H13 steel can be found in NADCA no. 229.

### 2.2. Specimen Dimensions

**Figure 1:**
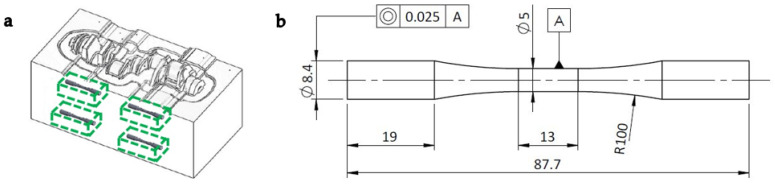
Origin of the steel specimens: (**a**) extraction of blocks from the crankshaft die and (**b**) specimen dimensions in millimetres after machining of the blocks.

Steel blocks were obtained from a crankshaft forging die, as represented in Figure 1a. These forging dies were already subjected to the heat treatment specified previously. Next, the specimens were machined from the steel blocks as per the geometry shown in Figure 1b, where the dimensions are in millimetres. The main axis of the specimens was aligned with the main axis of the crankshaft. These specimens were used for tensile and axial fatigue testing.

It should be pointed out that the specimen size can also affect the fatigue test results, as it raises the likelihood of presenting more and larger microstructural defects that may lead to premature crack initiation [28]. Multiple studies have been carried out where larger specimens showed significantly lower fatigue lives than smaller ones under the same loading conditions [29]. 

It is well known that surface roughness weakens fatigue strength as rough profiles present sharp stress concentrators that lead to premature crack initiation [30]. Therefore, the fatigue specimens were polished in order to remove any surface defects that may have originated throughout the manufacturing stage and to obtain a surface roughness below Ra = 0.2 µm.

### 2.3. Characterisation Methods

A microstructural analysis of the two studied steels was carried out on a JEOL JSM-6010 LA scanning electron microscope (SEM). This analysis was complemented with X-ray diffraction (XRD) to compare the diffraction patterns of both of the steels and to find any potential difference between their microstructures. The XRD analysis was performed using a PANalytical X’Pert Pro X-ray diffractometer by means of monochromatic Cu-Kα radiation (wavelength 1.54 Å) over the 2θ range of 25–130° with a step size of 0.02°. In order to enable a clear observation of the microstructure, samples of both of the steels were saw-cut using a refrigerant consisting of a mixture of oil and water so that heating and microstructural changes were prevented. Next, the steel samples were ground with silicon carbide sandpaper using water as a lubricant and then mirror-polished with 6 μm and 1 μm diamond suspensions. Finally, the samples were etched at room temperature with a Nital 5% solution for 22 s.

Surface microhardness measurements were taken from the same samples that were used for the microstructural analysis using an HMV-G21 Series micro Vickers hardness tester. The applied load was 4.903 N (500 g) for 10 s. The Vickers hardness test was repeated 10 times for each steel and the hardness was obtained as the average of all the measurements.

Tensile tests were performed using a universal testing machine with a load capacity of 250 kN in order to obtain the UTS and YS values for each steel. These tests were displacement-controlled at a rate of 5 mm/min until fracture of the specimens. The UTS was calculated as the maximum load reached during the test over the initial cross-section area of the specimen, whereas the YS was obtained as the normal stress that corresponded to a plastic normal strain of 0.2%.

**Figure 2:**
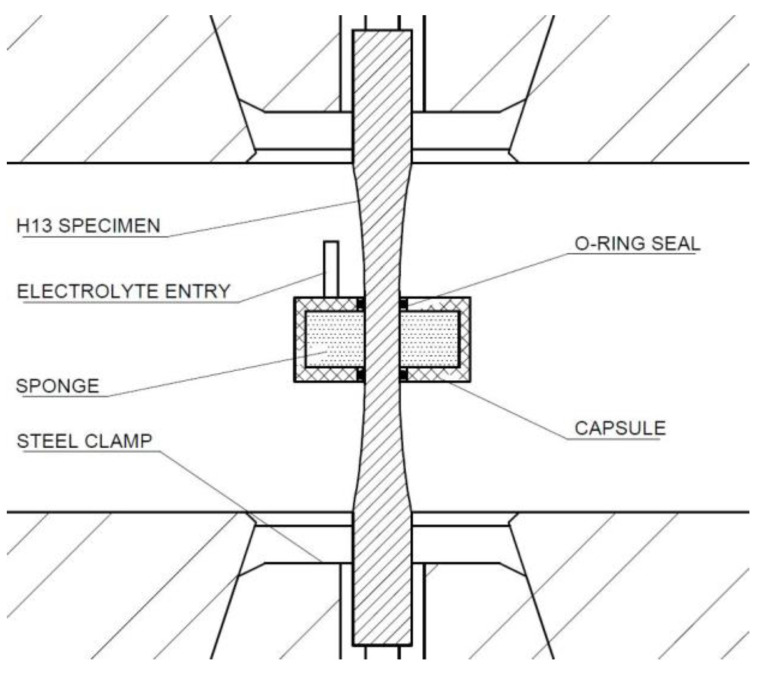
Schematics of the corrosion fatigue testing setup for surface soaking.

A Walter-Bai LFV-25 servo hydraulic dynamic test machine was used to perform the fatigue tests. This machine has a dynamic load capacity of ±25 kN, a maximum stroke of ±50 mm, a frame stiffness of 200 kN/mm, and a servo actuator accuracy of ISO 7500 class 0.5. This machine allows one to hold specimens with steel clamps hardened up to 60 HRC, which makes it suitable for testing hot forging tool steels. Load-controlled axial fatigue tests were carried out at room temperature according to the ISO 1099 standard [31]. In all the tests, the load profile was sinusoidal with a loading frequency of 10 Hz and a stress ratio equal to zero. In order to study the CF behaviour of these steels, the mid-section of the specimens was placed in contact with a sponge soaked with a corrosive medium throughout additional fatigue tests. The setup of these corrosion fatigue tests is shown schematically in Figure 2. The corrosive medium was a sodium chloride (NaCl) solution with a concentration of 0.1 M. All the specimens were tested at different load ranges up to either specimen failure or 10^7^ loading cycles (run-out). In the case of specimen failure, the output of the test was the number of cycles to failure. The load values applied to the specimens were selected to obtain fatigue lives within the HCF domain (10^4^–10^7^ cycles) under normal conditions. The same load values were applied to the specimens subjected to CF tests for the sake of comparison. The least squares method was applied for the data fitting of the fatigue results to obtain the Wöhler diagrams. As a result of all the combinations of the type of material (A-type and B-type steels) and test conditions (normal HCF and CF), four Wöhler diagrams were considered. 

Fracture surface images were obtained using a Nikon SMZ1000 microscope and a JEOL JSM-6010 LA SEM.

## 3. Results

### 3.1. Microstructure

**Figure 3:**
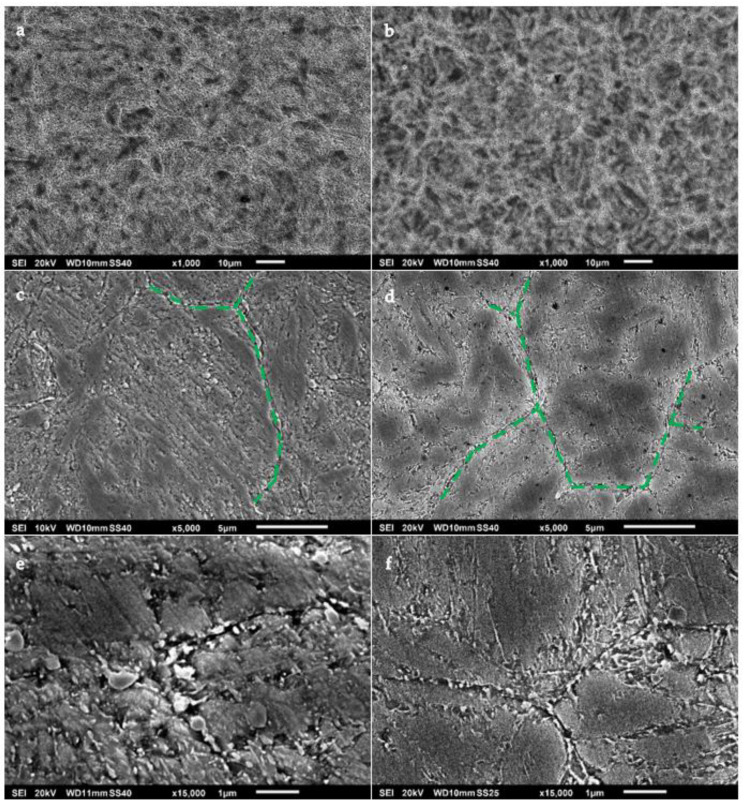
SEM images of (**a**,**c**,**e**) A-type and (**b**,**d**,**f**) B-type H13 steel microstructures at (**a**,**b**) ×1000, (**c**,**d**) ×5000, and (**e**,**f**) ×15,000.

The samples of both of the steels were taken using SEM analysis. Microstructure images of these samples are displayed in Figure 3 at different magnifications. These samples were mirror-polished and etched with an acid solution (Nital 5%) in order to obtain a clear observation of the microstructures. 

As can be seen in Figure 3a,b, the grain boundaries of the steels were revealed due to the acid attack. Furthermore, these images suggest that the grain size in the B-type steel was slightly smaller than that in the A-type steel. In Figure 3c,d, the typical martensitic laths with intermetallic carbides can be observed for both materials. Some grain boundaries are highlighted with dashed lines for clarity. The microstructural features of both materials resembled each other to some extent. However, the A-type steel showed a higher volume of carbides than the B-type steel. These carbides were significantly more abundant and coarser, and they adopted grain boundaries as their preferential position. The images shown in Figure 3e,f allowed us to confirm that the A-type steel was richer in primary carbides, whereas the B-type steel presented a lower number of primary carbides but a higher content of secondary carbides. The primary carbides had round and polygonal shapes, with sizes between 100 and 500 nm, whilst the secondary carbides were significantly smaller. The usual carbides observed in hot forging tool steels are chromium (M_7_C_3_ and M_23_C_6_), molybdenum (M_6_C), and vanadium (M_8_C_7_ and MC) [32]. 

As a result of the microstructural analysis, differences between the two studied steels were observed. A possible reason for the bigger grain size of the A-type steel could be due to the slightly higher austenitising temperature that this steel was subjected to. However, since the discrepancy between the austenitising temperatures of both steels was minimal, it seems more likely that different cooling rates were applied to the steels during quenching. Increasing the quenching cooling rate would cause a decrease in the average grain size, as well as a lower fraction and size of carbides. The reason for this phenomenon could be due to the insufficient time given to the carbides to coarsen at high cooling rates [33]. Therefore, it is likely that the B-type steel was subjected to a higher cooling rate than the A-type steel during the quenching stage of the heat treatment.

### 3.2. XRD

**Figure 4:**
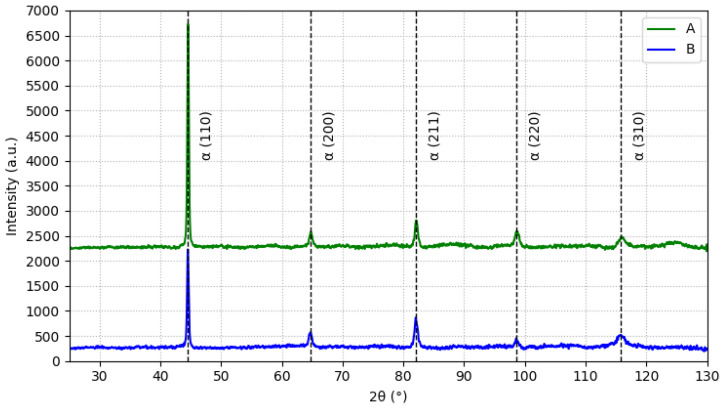
Diffractograms obtained from the XRD analysis of both H13 steels.

XRD analysis was carried out to identify the phases in both of the materials and to detect any potential differences between their microstructures. The results of the XRD analysis are displayed in Figure 4, where the crystallographic plane indexes are indicated for each diffraction peak. It was observed that both steels presented a characteristic diffractogram of a martensitic microstructure, with no signs of the austenitic phase [34,35]. Furthermore, the positions of the diffraction peaks matched perfectly, which unequivocally proved that both steels were constituted by identical phases. 

### 3.3. Hardness

Hardness measurements were obtained through Vickers tests for both of the steels. The hardness of the A-type steel was 455 HV, and for the B-type steel it was 458 HV, with combined standard uncertainties of 6 HV and 11 HV, respectively. Both materials showed mean values considerably close to 46 HRC (≈460 HV), as specified. No significant distinctions were observed between the measurements performed near the surface and in the interior of the samples. 

Both of the steels presented an equal hardness despite the different grain sizes and carbide distributions observed in the microstructural analysis. As a result of their equal hardness, both of the steels should have a similar resistance to crack initiation and should thus show comparably similar fatigue lives in HCF tests under normal conditions.

### 3.4. Tensile Tests

The specimens of both types of steel were subjected to tensile tests. The load applied to each specimen was recorded as a function of the axial displacement, which was increased at a rate of 5 mm/min during the test. The resultant UTS of each steel was calculated dividing the maximum load reached during the test over the minimum cross-section area of the specimen. The UTS of the A-type steel was 1470 MPa, whereas the UTS of the B-type steel was 1580 MPa, with combined standard uncertainties of 40 MPa and 50 MPa, respectively. Therefore, the mean UTS of the B-type steel was 7.5% higher than that of the A-type steel. The YS of each steel was obtained as the stress value that caused a plastic strain of 0.2%. The YS of the A-type steel was 1430 MPa, whereas the YS of the B-type steel was 1480 MPa, with combined standard uncertainties of 40 MPa and 50 MPa, respectively. In this case, the mean YS of the B-type steel was only 3.5% higher than the YS of the A-type steel. 

The modest differences in the UTS and YS values of the steels were consistent with the dissimilar grain sizes observed in the microstructural analysis as the B-type steel grains were found to be marginally smaller than those of the A-type steel. Despite these minor differences between the two types of steel, the results of these tensile tests demonstrated that the H13 steels were able to provide remarkable mechanical strengths.

### 3.5. Fatigue Tests

Axial fatigue tests were carried out at room temperature. All the tests were load-controlled using a sinusoidal waveform with a frequency of 10 Hz and a stress ratio equal to zero. The output of all the fatigue tests was either the number of cycles to failure or a run-out at 10^7^ cycles. Each specimen was subjected to a unique maximum stress selected so that the corresponding point in the Wöhler diagram fell within the HCF domain of the material. Likewise, the CF tests were developed under analogous conditions, although in these tests the surface of the specimen test section was placed in contact with a 0.1 M NaCl aqueous solution. Nine specimens of each steel were subjected to HCF tests, whereas six specimens of each steel underwent the CF tests. As a result of all the combinations of the type of material and test conditions, four datasets were considered.

The least squares method was applied to each of the four datasets in order to describe the HCF performance according to the Basquin exponential law for all the combinations of the type of steel and test conditions [36]. Each dataset was fitted to Equation (1):

(1) $$\sigma_{\mathrm{MAX}}=aN^{b},$$

where $\sigma_{\mathrm{MAX}}$ is the maximum stress applied on the fatigue test, N is the number of cycles to failure, a is a coefficient of fatigue strength, and b is the exponent of fatigue strength. As a result of this regression fit, the coefficients presented in Table 2 were obtained. The coefficients of determination $R^{2}$ were added to this table to evaluate the goodness of fit in each case.

**Figure 5:**
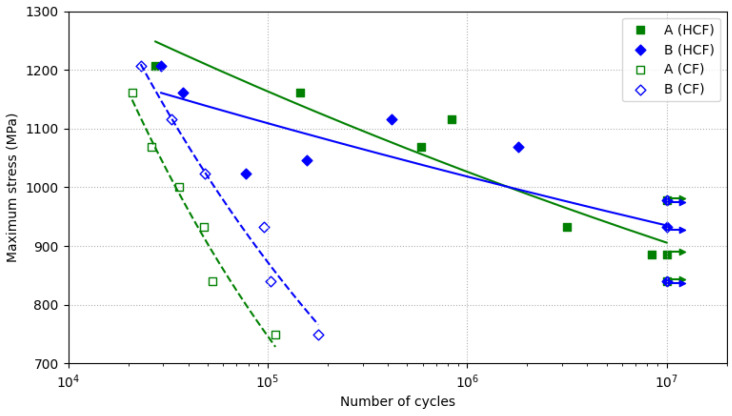
Wöhler diagrams obtained for the A-type and B-type H13 steels in the high-cycle fatigue domain under normal and corrosion fatigue conditions.

The results of all the fatigue tests are displayed in Figure 5 as Wöhler diagrams. In these diagrams, the maximum axial stress in MPa is plotted versus the number of cycles to failure of the specimen using a semi-logarithmic scale. The filled dots shown in the diagrams represent the specimens tested to HCF and the blank dots represent specimens that were tested to CF. Moreover, the green square-shaped dots denote the fatigue test results of the A-type steel specimens, whereas the blue diamond-shaped dots indicate the fatigue test results obtained from the B-type steel. Run-out tests are indicated with an arrow pointing rightwards at 10^7^ cycles. As for the tendency curves calculated in the regression analysis, the HCF curves are shown as continuous lines, whilst the CF curves are shown as dashed lines.

As can be observed in Figure 5, the fatigue behaviour under normal conditions was similar for both H13 steels. Both steels were able to resist 10^7^ loading cycles when they were subjected to a maximum stress of 980 MPa (68% and 66% of the YS of the A-type and B-type steel, respectively). Nevertheless, the fact that few A-type steel specimens failed below that stress, while B-type steel specimens did not, suggests that the actual fatigue limit of the A-type steel could be marginally lower than that of the B-type steel. The potentially better fatigue limit of the B-type steel would be in good agreement with the microstructural analysis carried out in this work, as the A-type steel showed a higher abundance of coarser carbides than the B-type steel. Finer carbides should hinder crack propagation, whereas coarser carbides and inclusions help promote crack initiation and are detrimental to fatigue behaviour [17].

The scatter of the HCF results obtained here could be attributed to the bimodal distribution of fatigue life in the transition between HCF and very-high-cycle fatigue (VHCF) domains. This transition between domains extends within a certain range of stress of the material. Within this range of stress, the probability of fracture in the HCF domain decreases as the applied load decreases [37]. Moreover, microstructural heterogeneities and a wide distribution of defects, such as coarse carbides and inclusions, may raise the scatter of fatigue results within the transition between HCF and VHCF domains [38].

The methodology utilised in this study to develop the CF tests, despite being rather simple, proved to be effective and economical. An abrupt decrease in fatigue lives in the CF tests as compared with the HCF tests was perceptible. Moreover, this difference between the fatigue lives in the test conditions was more noticeable at lower stress values than at higher ones. As a result of these differences, the exponents of the fatigue strength calculated for the CF tests were much lower (more negative) than those of the datasets obtained from the HCF tests. Lower exponents of fatigue strength resulted in steeper Wöhler diagrams. Therefore, no signs of fatigue limit were observed for the CF tests within the stress levels that they were subjected to in this study. All of the mentioned observations of the CF curves were in accordance with the contributions of other researchers [39,40]. 

The CF lives of the B-type steel were approximately twice as long as those of the A-type steel. Unlike the HCF tests, the Wöhler diagrams of both steels obtained from the CF tests remained virtually parallel and did not overlap. It should also be pointed out that the data were much less scattered for the CF tests than for the HCF tests. The coefficients of determination, displayed in Table 2, were higher than 0.95 for the CF tests, whereas they did not exceed the value of 0.85 for the HCF tests. It was not surprising that the data scatter was reduced in the CF tests since the mechanism that triggered fatigue failure in the CF tests was the formation of corrosion pits rather than microstructural features. The corrosion pits acted as sharp stress concentrators that eventually opened a crack in the surface of the specimen that was subjected to fatigue loads. In contrast, crack initiation in HCF tests was attributed to the presence of surface defects and inclusions, so crack initiation required more time to be developed. Therefore, the presence of microstructural defects and inclusions seemed to play a minor role in the CF tests as compared to the HCF tests as the specimen failure was advanced substantially in the CF tests. In order to further understand these results, an analysis of the fracture surfaces of the specimens that failed in the fatigue tests was carried out.

### 3.6. Fracture Analysis

Fracture surface analysis was carried out on several specimens that failed after the fatigue tests. First, images of the whole fracture surfaces were taken using a stereoscopic microscope. Next, a deeper fracture surface analysis was performed using SEM. SEM is a powerful tool with a great depth of focus which permitted us to obtain good-quality images at a high magnification.

**Figure 6:**
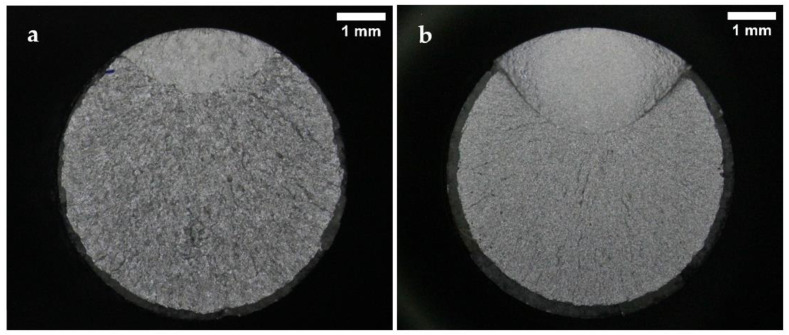
Fracture surfaces after fatigue tests of (**a**) A-type and (**b**) B-type H13 steels, tested in air to a maximum stress of 1200 MPa (84% and 81% YS, respectively).

**Figure 7:**
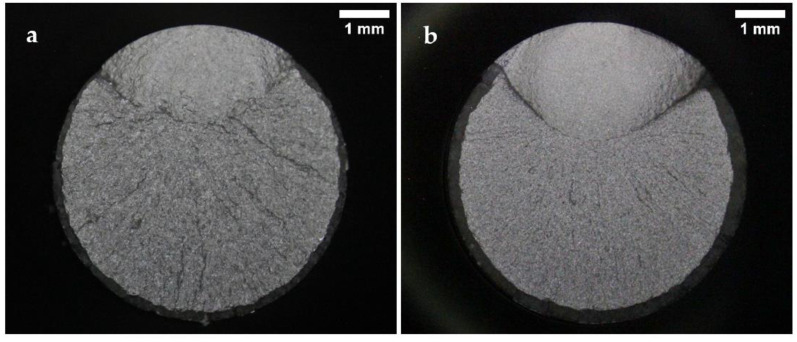
Fracture surfaces after fatigue tests of (**a**) A-type and (**b**) B-type H13 steels, tested in air to a maximum stress of 1160 MPa (81% and 78% YS, respectively).

**Figure 8:**
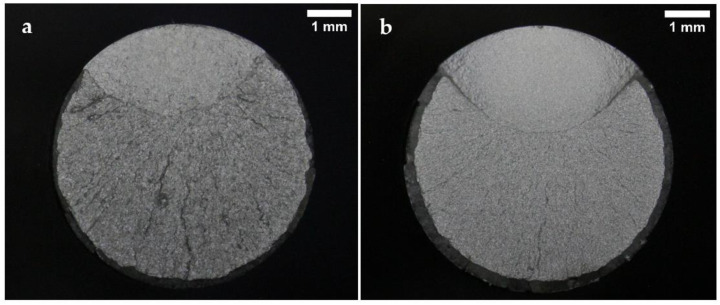
Fracture surfaces after fatigue tests of (**a**) A-type and (**b**) B-type H13 steels, tested in air to a maximum stress of 1070 MPa (75% and 72% YS, respectively).

Representative fracture surface images of the specimens tested under normal conditions are presented in Figure 6, Figure 7 and Figure 8. The images show the characteristic zones of a typical axial fatigue fracture with no stress concentration [41], namely, the fatigue crack propagation zone, the final fracture zone, and the shear lip. The remarkable symmetry and crack directionality of these fracture surfaces confirmed that crack initiation took place at a single point of the surface of the specimens. After the crack was opened, it started growing at a rate dependent on the applied stress intensity range. Once the crack reached a size such that the remaining cross-section was no longer able to support the applied load, a sudden fracture of the specimen occurred. Therefore, it was not surprising that, for lower stress amplitude values, the fatigue crack propagation area increased in size whilst the final fracture area decreased. As a result of the overall fracture development process, the fatigue crack propagation zones were comparatively smoother than the final fracture zones. The shear lips indicated the ultimate failure location of the specimens, and they extended throughout the perimeter of the final fracture areas.

Some differences between the fracture surfaces of both steels were observed. It was noted that for the same applied stress range, the size of the crack propagation areas and the shear lip widths were greater in the B-type steel than in the A-type steel. This difference in the crack propagation areas was in line with the results obtained in the tensile tests, as the B-type steel presented higher YS and UTS values than the A-type steel. Under the same fatigue test conditions, the material with higher UTS is expected to present a lower final fracture area. Moreover, the B-type steel fracture surfaces looked smoother than those of the A-type steel. The distinctions observed in the fracture surface roughness of both steels were attributed to their dissimilar microstructures, since the B-type steel proved to have a lower grain size and finer carbides than the A-type steel.

**Figure 9:**
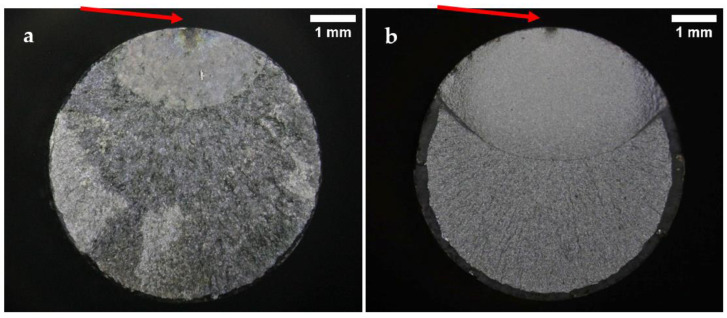
Fracture surfaces after fatigue tests of (**a**) A-type and (**b**) B-type H13 steels, tested in a corrosive medium to a maximum stress of 750 MPa (52% and 51% YS, respectively).

All the corrosion fatigue specimens failed due to surface crack initiation. The fracture surfaces of two specimens from the corrosion fatigue tests are shown in Figure 9. These specimens were tested to a maximum stress of 750 MPa (52% and 51% of the YS of A-type and B-type steel, respectively), which means that they would have been run-outs if they were tested under normal conditions. Essentially, these images revealed almost the same features as those of normal fatigue tests. However, it was noted that failure took place due to the development of a corrosion pit on the surface of the specimens. The corrosion pits are indicated with arrows in Figure 9. The formation of these corrosion pits accelerated the crack initiation process and thus decreased the fatigue lives of these specimens dramatically. In this case, the size of the crack propagation area was significantly greater in the B-type steel than in the A-type steel.

**Figure 10:**
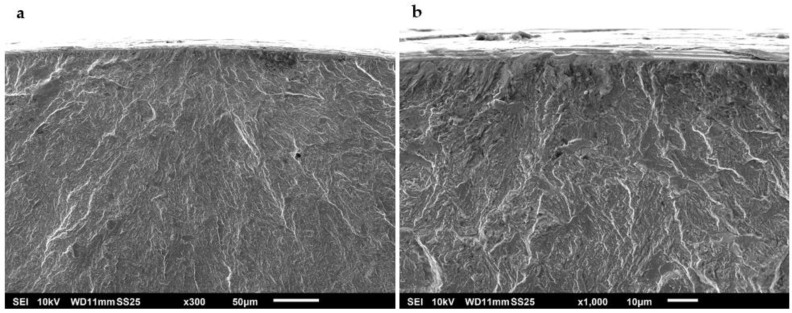
Surface crack initiation of an A-type steel specimen tested in air to a maximum stress of 1070 MPa (75% YS) at (**a**) ×300 and (**b**) ×1000.

**Figure 11:**
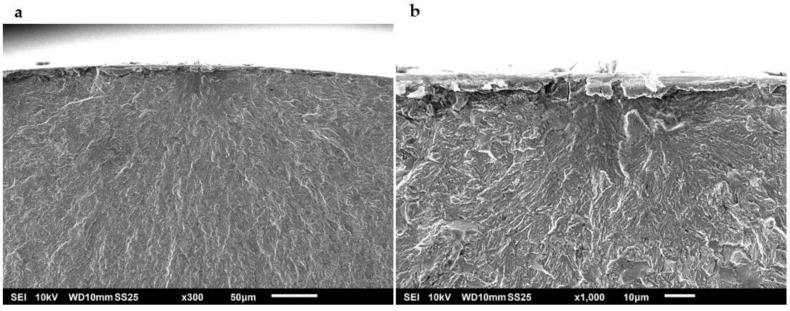
Surface crack initiation of a B-type steel specimen tested in air to a maximum stress of 1070 MPa (72% YS) at (**a**) ×300 and (**b**) ×1000.

Fracture surface images with a high magnification were obtained using SEM. Under normal test conditions, most of the specimens revealed that crack initiation took place at a single point on the surface of the specimens. Examples of specimens that presented surface crack initiation are shown in Figure 10 and Figure 11. The predominant mechanism of the crack initiation region was a transgranular fracture. Crack initiation was likely to occur in a surface defect of these specimens, such as a small scratch or an indentation that acted as a notch and raised the stress locally. Intermetallic carbides could have also intervened as stress concentrators that triggered crack initiation in these steels as they are heterogeneities present in the material [42]. Crack opening was driven by shear stress at this point. After the crack reached a size of a few micrometres, it started to propagate perpendicularly to the tension load applied to the specimen [43]. Underneath the surface, even though the fractures were predominantly transgranular, signs of intergranular fracture could be observed at higher magnifications.

**Figure 12:**
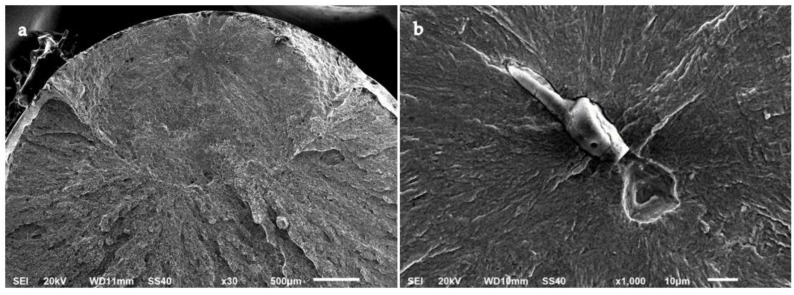
Internal crack initiation and fish-eye region of an A-type steel specimen tested in air to a maximum stress of 1110 MPa (78% YS) for 833,119 cycles at (**a**) ×30 and (**b**) ×1000.

**Figure 13:**
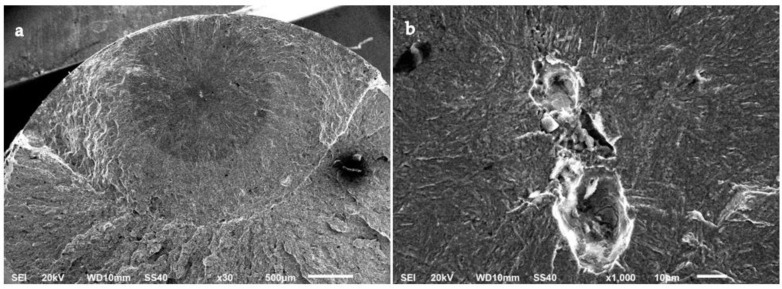
Internal crack initiation and fish-eye region of an A-type steel specimen tested in air to a maximum stress of 890 MPa (62% YS) for 8,370,000 cycles at (**a**) ×30 and (**b**) ×1000.

Unlike most of the tested specimens, two A-type steel specimens failed due to internal crack initiation. The fracture surfaces of these specimens are shown in Figure 12 and Figure 13. In these specimens, crack initiation occurred at an inner point of the material and extended radially. Furthermore, a circular dark area can be distinguished in Figure 12a and Figure 13a, which is commonly known as the fish-eye region. At the centre of these circular areas, the presence of an inclusion or a cluster of inclusions was revealed. The size of these inclusions was greater than 10 µm, and they might have led to premature crack initiation on the sample. These inclusions were mainly oxides of calcium, magnesium, and aluminium from the electrode, which survived the ESR manufacturing process due to their high melting point [44]. No internal crack initiation or inclusions were observed in the B-type steel specimens.

On the fracture surfaces analysed in this study, the so-called fish-eye morphology was present, but no fine granular area (FGA) was observed [45]. The darkness of the fish-eye was ascribed to the absence of air contact with the fracture surface until the crack reached the specimen surface [46]. Moreover, the size of the fish-eye area was strongly correlated with the depth of the inclusion that initiated the main crack [47], which is in accordance with the observations shown in Figure 12 and Figure 13.

The likelihood of internal crack initiation depends on the applied stress range and the average size of inclusions. When these inclusions reach a size of several micrometres, they can be treated as effective cracks [48]. At lower stress values, the stress concentration due to the surface defects may not be as high as at inclusions, so the likelihood of internal crack initiation is enhanced. In fact, mathematical models have been proposed that estimate the fatigue strength of steels in the VHCF regime using the inclusion area as one of the main parameters of the model [49]. Even though internal crack initiation is a usual fracture mechanism of the VHCF regime, internal crack initiation has also been observed at cycles to failure as low as 10^5^ cycles [50].

**Figure 14:**
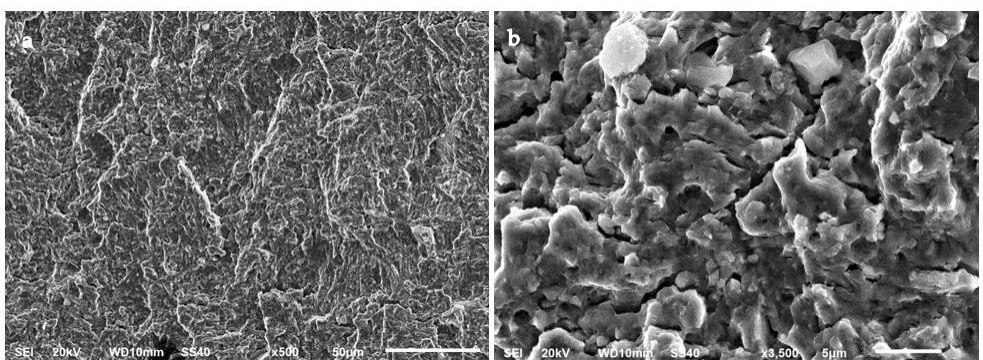
Crack propagation area of an A-type steel specimen tested in air to a maximum stress of 1160 MPa (81% YS) at (**a**) ×500 (**b**) ×3500.

**Figure 15:**
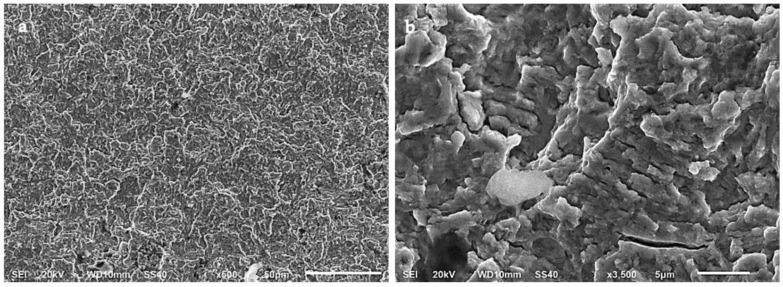
Crack propagation area of a B-type steel specimen tested in air to a maximum stress of 1020 MPa (69% YS) at (**a**) ×500 and (**b**) ×3500.

Plastic deformation in the stable crack growth area of an A-type steel and a B-type steel is shown in Figure 14 and Figure 15, respectively. Striations of less than 1 µm in width can be distinguished in these specimens, which revealed the slip plane during crack propagation. These striations were often located in individual grains within a fracture surface caused by transgranular plastic deformation. The width of the striations can be associated with the crack growth rate during the fatigue test. Secondary cracks can also be seen in the fracture surfaces, usually perpendicular to the direction of the main crack propagation. These secondary cracks are also evidence of a high crack propagation rate. Furthermore, it was observed that the main crack propagated through non-coplanar grains. 

The SEM images also revealed a more gradual transition of the crack propagation through non-coplanar grains in the B-type steel (Figure 15a) as compared to the A-type steel (Figure 14a). Intermetallic carbides could have acted as obstacles for crack propagation [17], deviating the main crack to different non-coplanar grains during crack growth. Therefore, the presence of coarser carbides in the A-type steel specimens would explain why the fracture surfaces of these specimens were rougher than those of the B-type steel.

In summary, fracture surface analysis was carried out to further understand and explain the results obtained from the fatigue tests of hot forging tool steels. Most specimens presented a unique crack initiation site located on the surface. Moreover, it was demonstrated that a few A-type steel specimens failed due to internal cracks originating at inclusion locations. The sizes of the crack propagation areas of the B-type steel were significantly lower and smoother than those of the A-type steel. These differences were attributed to the coarser carbides observed in the A-type steel through the microstructural analysis. Overall, these observations suggest that the more homogeneous microstructure of the B-type steel provided a higher resistance to fracture than the A-type steel in the presence of cracks.

## 4. Discussion

In the present work, the axial fatigue behaviour within the HCF domain of two H13 steels has been studied. As a novel contribution, the CF results of these H13 steels have been analysed for the first time. On the one hand, a microstructural analysis was carried out prior to the fatigue tests. The microstructural analysis revealed some dissimilarities between the steels studied here regarding their grain sizes and carbide distributions. These dissimilarities anticipated potential differences in the fatigue behaviour of both materials, despite belonging to the same steel specification. On the other hand, the phase compositions, hardness, and the specimens utilised in the fatigue tests were identical for both steels. Hence, this work has allowed us to analyse the effect of some microstructural features on the fatigue behaviour of hot forging tool steels under different work environments. 

Both steels presented reasonably similar results in the HCF tests under normal conditions. The HCF results were presented as the maximum stresses supported in axial tests when the stress ratio was zero. If these results were converted to stress amplitudes, the resultant values would be approximately 75–85% of those reported by Shinde et al. [17] for H13 or by Korade et al. [18] for H21 in rotating bending tests. This proportion aligns with the experimental differences observed between the axial and bending fatigue tests. The different results of both of the test methodologies are due to the fact that a higher proportion of the specimen’s volume is subjected to the maximum stress in the axial fatigue tests as compared to the rotating bending fatigue tests [51]. 

The corrosion fatigue lives of the H13 steels were dramatically lower than the fatigue lives obtained under normal conditions. Moreover, this reduction in the fatigue life results was more evident as the applied stress range was decreased. At higher stress levels, the mechanical damage due to the applied stress was much higher than the corrosion damage; thus, the fatigue lives were found to be similar regardless of the test environment. At lower stress levels, corrosion damage was reported to overcome mechanical damage as the formation of corrosion pits takes place [21]. The corrosion fatigue behaviour of the H13 steels observed in the present study was in good agreement with the findings of other studies regarding the corrosion fatigue of martensitic steels [22,23]. 

Interestingly, the coefficients of determination obtained for each material and test condition were higher for the CF tests than for the HCF tests. These results meant that the data scatter was greater in the conventional HCF tests. In the conventional HCF tests, microstructural defects are known to be an important source of scatter [38]. Crack initiation is appreciably sensitive to the size distribution of defects and the microstructural heterogeneities of the specimen. Under such conditions, the time invested in crack initiation represents almost the totality of the fatigue lives in the HCF domain. 

In contrast, crack initiation occurred due to the formation of corrosion pits in the CF tests. The formation of corrosion pits advanced the failure of specimens substantially. The loading frequency plays a key role in the development of corrosion pits. Lower loading frequencies proved to result in lower corrosion fatigue lives [21]. If two specimens were subjected to CF tests at different loading frequencies, the one with the lower frequency would undergo a lower number of loading cycles than the other at the same time instants. The fatigue lives of the specimens in the CF tests should depend strongly on the time required to develop a corrosion pit, as corrosion pits are the mechanisms that trigger crack initiation in these tests. The kinetics of corrosion pit formation depend mostly on the loading frequency [52], the salt concentration [53], the temperature, and the pH of the corrosive medium [54]. Since all these variables remained identical for all the specimens, the crack initiation times of the CF tests are believed to have been virtually alike. As a result, similar crack initiation times led to lower data scatter. 

Another feature that should be emphasised is the difference observed between the crack propagation areas of both the steels when they were tested at the same stress level. The A-type steel presented smaller crack growth regions than the B-type steel. Furthermore, this difference between crack growth regions was more remarkable at lower stresses. This observation suggested that, for a certain crack size, the B-type steel was able to support a higher load than the A-type steel. The fact that the B-type steel showed marginally higher strength than the A-type steel accords with the tensile test results. In the tensile test results, the B-type steel had a UTS that was 7.5% higher and a YS 3.5% that was higher than those of the A-type steel. Nevertheless, this discrepancy between the YS and UTS values did not seem significant enough to explain the large difference between the crack growth region sizes of both steels at low stress ranges (see Figure 9). These different crack propagation sizes suggested that the B-type steel had higher fracture toughness than the A-type steel, as fracture toughness characterises the material’s resistance to crack propagation.

The potentially higher fracture toughness of the B-type steel could also explain its better performance as compared to the A-type steel in the CF tests. It has been stated that all the CF tests should reveal similar crack initiation times as this time depends on the kinetics of the development of corrosion pits. The fact that the B-type steel presented fatigue lives that were approximately twice as high as those of the A-type steel in all the CF tests should be attributed to longer crack propagation times. The longer crack propagation times of the B-type steel specimens would also align with the larger extension of their crack propagation areas. Moreover, the presence of coarser carbides and inclusions in the A-type steel is believed to have accelerated the fractures [17].

The mechanical properties of hot forging tool steels are strongly controlled by the parameters of the heat treatments that these steels are subjected to. Such mechanical properties include the UTS, hardness, and fracture toughness [3,4,12,13,14]. These properties are a consequence of the grain size and carbide distribution obtained at the end of the heat treatment. Slight discrepancies in the austenitising or tempering temperatures, times, or heating/cooling rates could have been factors that led to the dissimilar microstructures of the two types of steel. In this study, it is likely that the B-type steel was subjected to a higher cooling rate than the A-type steel during the quenching stage of the heat treatment, as this fact would explain the lower grain size and finer carbides of the B-type steel.

In summary, hot forging tool steels present outstanding static and dynamic mechanical properties. Nevertheless, an aggressive environment may result in the premature failure of these steels under cyclic loads. We encourage the study of the crack initiation and propagation phenomena of hot forging tool steels under different working environments in future research. Understanding the fatigue fracture mechanisms of these steels is crucial to guaranteeing the correct design of hot forging dies, thereby also saving resources invested in the maintenance of such tools. 

## 5. Conclusions

For the first time, this study presents the comparative results of H13 steels subjected to axial high-cycle fatigue tests under normal conditions and corrosion fatigue tests. The understanding of these damage processes in hot forging tool steels is fundamental to ensuring the extensive lives of forging tools, especially under aggressive environments.

The two H13 steels studied here, namely, the A-type and the B-type, were found to have identical microstructural phases and hardness values, with only slight differences in their YS and UTS values. Nevertheless, the B-type steel had a lower grain size than that of the A-type steel. Moreover, carbides in the A-type steel were coarser and more abundant than those in the B-type steel. These discrepancies were attributed to marginal variations in the heat treatments of both materials. 

The results of all the axial fatigue tests were fitted to the Basquin equation to mathematically represent the relationship between the maximum stress of the specimens, $\sigma_{\mathrm{MAX}}$, and the number of cycles to failure, N, for each material and test condition. As a result, the fatigue behaviours of the A-type and B-type steel under normal conditions were modelled by $\sigma_{\mathrm{MAX}}=2240.0\;N^{-0.057}\;\left(R^{2}=0.850\right)$ and $\sigma_{\mathrm{MAX}}=1711.7\;N^{-0.038}\;\left(R^{2}=0.673\right)$, respectively, whereas their corrosion fatigue behaviours were modelled by $\sigma_{\mathrm{MAX}}=16,662.6\;N^{-0.269}\;\left(R^{2}=0.964\right)$ and $\sigma_{\mathrm{MAX}}=11,808.1\;N^{-0.227}\;\left(R^{2}=0.971\right)$, respectively. 

Both of the H13 steels showed outstanding fatigue strengths in conventional high-cycle fatigue tests, reaching values of maximum stress as high as 980 MPa at 10^7^ cycles. However, the corrosion fatigue lives of the two types of steel were much lower than the conventional high-cycle fatigue lives, showing no signs of fatigue limit for the same range of applied stress. The significant decrease in the exponents of fatigue strength in the corrosion fatigue tests demonstrated that the effect of the corrosive agent on the fatigue lives was more noticeable at lower stress values. Furthermore, the coefficients of determination for the Basquin equations were much higher in the CF tests than in the HCF tests, which indicated a lower tendency of data scatter in the CF tests. The crack initiation stage in the conventional HCF tests involved greater randomness as it depended strongly on the size and distribution of the microstructural defects. In contrast, it is believed that the crack initiation times were similar in all the CF tests, as they mainly relied on the time required to create a corrosion pit due to exposure of the steels to the corrosive medium. 

Interestingly, both of the steels presented similar fatigue behaviour under the conventional HCF tests, whereas their CF lives were significantly different. The corrosion fatigue lives in the B-type steel were twice as high as those in the A-type steel. This difference between the corrosion fatigue lives was in line with the greater extension of the crack propagation regions in the B-type steel as compared to the A-type steel. Overall, the higher corrosion fatigue lives of the B-type steel can be attributed to its higher resistance to crack propagation.

The results obtained in the present study have emphasised the important influence of the steel microstructure on the fatigue behaviour and fracture toughness. The specimens with coarser carbides and higher grain sizes were found to have lower fracture strengths during crack propagation. The effect of fracture toughness on the steel durability was more noticeable in the CF tests than in the conventional HCF tests. Therefore, we propose analyses of the crack growth rate of hot forging tool steels under different aggressive environments as worthwhile research to be carried out in the future.

## Figures and Tables

**Table 1:** Chemical compositions of the two H13 steels in weight percentages.

	Name	C	Si	Mn	P	S	Cr	Mo	V	Fe
H13 steel	A	0.40	1.01	0.36	0.012	0.0020	5.20	1.31	0.95	Balance
	B	0.39	1.01	0.38	0.013	0.0005	5.11	1.43	0.92	Balance

**Table 2:** Coefficients obtained from the regression analysis of the fatigue tests.

Material	Condition	a [MPa]	b [-]	R^2^
A	HCF	2240.0	−0.057	0.850
	CF	16,662.6	−0.269	0.964
B	HCF	1711.7	−0.038	0.673
	CF	11,808.1	−0.227	0.971

## Data Availability

Not applicable.
